# Advances in Toughening Modification Methods for Epoxy Resins: A Comprehensive Review

**DOI:** 10.3390/polym17091288

**Published:** 2025-05-07

**Authors:** Jiawei Zhang, Zhen Zhang, Ran Huang, Lianjiang Tan

**Affiliations:** 1 School of Materials Science and Engineering, Shanghai Institute of Technology, Shanghai 201418, China; 236081279@mail.sit.edu.cn; 2 Shanghai Municipal Engineering Design Institute (Group) Co., Ltd., Shanghai 200092, China; zhangzhen8@smedi.com; 3 Institute of Biomedical Engineering and Technology, Fudan University, Shanghai 200433, China; 4 Yiwu Research Institute, Fudan University, Yiwu 322099, China; 5 Zhuhai Fudan Innovation Research Institute, Zhuhai 519031, China

**Keywords:** epoxy resin, toughness, modification

## Abstract

This work provides a comprehensive review of the recent advancements in the toughening modification methods for epoxy resins. The study explores a variety of approaches, including the incorporation of liquid rubbers, core–shell rubber particles, thermoplastic resins, hyperbranched polymers, and the nanoparticle toughening method, each of which contributes to improving the mechanical properties and fracture toughness of epoxy resins. Special attention is given to the mechanisms underlying these toughening methods, such as reaction-induced phase separation, crack pinning, and energy dissipation through particle deformation. The paper also examines the synergistic effects achieved by combining different toughening agents, such as phenoxy thermoplastic rubber and core–shell rubber particles, which significantly enhance the critical fracture energy and impact strength of epoxy composites. Additionally, the challenges associated with each method, such as the potential reduction in mechanical properties and the influence of phase separation on material performance, are discussed. Through a detailed analysis of experimental studies, this paper highlights the effectiveness of various toughening strategies and suggests future research directions aimed at further optimizing epoxy resin toughening techniques for diverse industrial applications. Emerging computational modeling and machine learning applications in epoxy resin development are also systematically reviewed to highlight their potential in advancing predictive design frameworks.

## 1. Introduction

**Figure 1:**
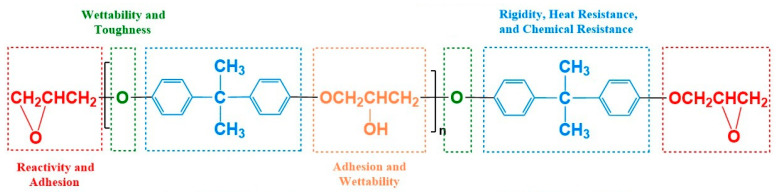
Chemical structure of bisphenol type A epoxy resin.

Epoxy resin (EP) is one of the most widely consumed thermosetting resins among major commercial resins, distinguished by its extensive variety, flexible formulation design, and broad application range [1]. Epoxy resins are high-performance thermosetting polymers characterized by the presence of two or more epoxy groups within their molecular structure, typically based on aliphatic, cycloaliphatic, or aromatic carbon chains [2,3,4] (Figure 1). The epoxy groups (-C_2_H_4_O-) at a molecular level provide notable reactivity and exceptional adhesive properties to the resin. The presence of the benzene ring structure adds high rigidity, heat resistance, and chemical stability, guaranteeing that cured epoxy resins maintain consistent mechanical properties even at high temperatures. Moreover, the inclusion of ether bonds (-O-) and hydroxyl groups (-OH) improves the resin’s capacity to be wetted, hence promoting stronger chemical interaction with the surfaces of substrates. Hydroxyl groups facilitate hydrogen bonding with other molecules, hence enhancing intermolecular interactions and overall material performance. Epoxy resins has exceptional properties such as strong adhesion, resistance to wear, effective electrical insulation, chemical stability, capacity to withstand high and low temperatures, dimensional stability, ease of processing, and affordability. These qualities make epoxy resins very versatile and suitable for use in a wide range of industries and applications [5,6,7].

Due to the presence of chemically reactive epoxy groups in its molecular structure, epoxy resin can undergo crosslinking reactions with various types of curing agents, resulting in a highly crosslinked three-dimensional network structure after curing [8]. While this structure imparts excellent performance characteristics, it also leads to significant brittleness, poor impact resistance, and a tendency to crack [9,10]. These shortcomings have notably limited the application of epoxy resins in fields requiring high impact and fracture strength [11,12].

The introduction of toughening agents can significantly enhance the performance of epoxy resins. Specifically, toughening agents can markedly improve the resin’s toughness, reducing or preventing cracking. Additionally, they effectively increase the impact resistance of epoxy resins, making them more resilient to external shocks and vibrations. The incorporation of toughening agents also improves the processing and molding properties of epoxy resins, enhancing the flexibility of manufacturing processes. Toughened resins typically exhibit higher tensile and flexural strengths, providing more reliable and stable performance for structural and engineering applications.

Many researchers have developed and studied various types of epoxy toughening agents to achieve diversified performance enhancements. Among these, the rubber toughening method, which introduces rubber particles, significantly improves the impact resistance of epoxy resins. The thermoplastic resin toughening method effectively enhances the material’s plasticity and reprocessability. Nanoparticle toughening relies on nanoscale particles to strengthen the material’s durability and wear resistance. Hyperbranched polymer toughening strengthens the molecular network through a hyperbranched structure, enhancing intermolecular interactions. Flexible chain segment toughening increases the flexibility of molecular chains, reducing rigid constraints between them. Table 1 provides a detailed comparison of the advantages and disadvantages of these toughening methods.

Through an in-depth review of domestic and international literature, this paper comprehensively summarizes the mechanisms and latest research advancements in the toughening modification of epoxy resins using various methods such as rubber toughening, thermoplastic resin toughening, hyperbranched polymer toughening, and flexible chain segment toughening. Following the systematic exposition of conventional experimental development and testing methodologies, this study provides a comprehensive synthesis of computational modeling techniques and machine learning applications in epoxy resin development and performance prediction. Additionally, the paper provides an outlook on future research directions, aiming to offer a more comprehensive and timely understanding of the developments in this critical field.

## 2. Rubber Toughening

Rubber toughening of epoxy resins is one of the earliest toughening methods, and the research on this technique is relatively mature [21]. Rubber toughening is characterized by its simple preparation process and low cost, making rubber the most extensively studied and widely used material for toughening epoxy resins. Researchers have continually optimized the toughening effect by selecting, synthesizing, and characterizing various types of rubber elastomers. As a result, rubber-toughened epoxy resins have found widespread applications in fields such as aerospace, automotive manufacturing, and composite materials.

### 2.1. Mechanisms of Rubber Toughening

The mechanisms of rubber toughening in epoxy resins are diverse, often involving multiple mechanisms working in conjunction. The two primary mechanisms are shear yielding theory and cavitation theory.

#### 2.1.1. Shear Yielding Theory

In the 1960s, Newman and colleagues, through the study of the deformation characteristics of rubber elastomers in ABS, found that rubber elastomers could significantly reduce the yield stress of materials and promote shear yielding. This led to the proposal that the toughening of the matrix resin by rubber elastomers is primarily due to the shear yielding of the matrix [22]. They posited that rubber elastomer particles act as stress concentrators within the matrix resin. When subjected to stress, these particles cause cavitation in the matrix, interface debonding, and the formation of crazes, which lower the glass transition temperature and make the matrix more prone to plastic deformation, thereby achieving toughening.

Subsequently, Bucknall and others [23,24] observed in experiments that shear bands passed through rubber particles and that numerous crazes were terminated by these shear bands. This observation led to the conclusion that the combined action of crazing and shear bands is the primary mechanism for material toughening. Based on these findings, Wu and colleagues [25,26] proposed that the stress induced by crazing in the matrix is proportional to the square root of the chain entanglement density, while the critical yield stress of the matrix is positively correlated with the characteristic ratio of unperturbed chains. From these relationships, it was deduced that, when the stress induced by crazing in the matrix is below its critical yield stress, the crazing mechanism dominates the toughening process. Conversely, if the crazing stress exceeds the critical yield stress, the shear yielding mechanism becomes the primary mode of action.

#### 2.1.2. Cavity Theory

In the 1980s, Pearson et al. [27] proposed that the formation of cavities, or voids, at the interface between rubber elastic particles and the matrix is a primary mechanism for toughening epoxy resin systems. When subjected to external forces, dispersed rubber particles create stress concentration effects, which generate three-dimensional tensile stresses in the surrounding matrix and induce cavitation in the rubber particles, leading to the formation of voids or holes around them. This cavitation process causes debonding at the particle–matrix interface, allowing the rubber particles to release their inherent elastic strain energy and thus contribute to toughening. Additionally, cavitation can promote shear yielding in the material, suppress crack propagation, and dissipate substantial amounts of energy, resulting in increased toughness [28].

### 2.2. Factors Influencing Rubber Toughening

The factors affecting the toughening effect of rubber are complex and multi-faceted. In addition to key parameters such as rubber particle size, content, and distribution, the inherent properties of the epoxy resin, the interface conditions between the matrix and the dispersed phase, and their compatibility also significantly impact the rubber toughening effect.

#### 2.2.1. Rubber Content

Rubber content is a primary factor influencing the toughening effect. Generally, the toughening effect increases with rubber content up to a certain point, after which it decreases. He et al. [29] investigated the effect of rubber content on the fracture behavior of toughened epoxy resins. To alter the concentration without changing other morphological characteristics, they dispersed preformed acrylic particles in liquid epoxy resin monomer to prepare the material. The study indicated that toughness increased with rubber content, reaching a maximum value between 12.5 phr and 25 phr, and remained relatively constant. However, further increases in rubber concentration led to a gradual decrease in toughness, with crack propagation behavior transitioning from unstable to stable.

Similarly, N. Chikhi et al. [30] incorporated varying amounts of acrylonitrile–butadiene–styrene (ABS) copolymer with 16% acrylonitrile content into bisphenol-A-type epoxy resin cured with multi-amino imidazoline to enhance toughness. Their research showed that impact strength and fracture toughness (K_IC) reached maximum values when the ATBN content was 12.5 phr. Therefore, for a specific rubber-toughened epoxy resin system, there exists an optimal rubber content that maximizes the toughening effect.

#### 2.2.2. Rubber Particle Size

Within a certain size range, smaller rubber particles often exhibit better dispersion properties and are more effective at initiating silver lines and shear yielding, leading to improved toughening performance. However, smaller particles are not always better. Day et al. [31] investigated the effect of particle size on the impact toughness of piperidine-cured epoxy resins. Their study found that the particle size around the rubber layer needs to be greater than 0.35 µm; reducing the particle size around the rubber layer results in decreased fracture toughness.

Bagheri et al. [32] incorporated rubber particles of various sizes and hollow latex particles into DGEBA epoxy resins. Their research indicated that within the range of 0.2 to 40 µm, smaller particles generally result in higher fracture toughness. Pearson et al. [33] suggested that the optimal rubber particle size for toughening depends on the properties of the epoxy resin but typically falls within the range of 0.1 to 5 µm. Particles smaller than 0.1 µm are too small to effectively cavitate and thus do not contribute to the toughening process. Particles larger than 5 µm primarily act as bridging particles, as they do not interact with the stress field at the crack tip.

#### 2.2.3. Properties of Epoxy Resins

The toughening effect is closely related to the inherent properties of the epoxy resin, with crosslink density being a particularly important factor. Pearson et al. [27] investigated the impact of crosslink density on the toughening of epoxy resins by using diglycidyl ether of bisphenol A (DGEBA) epoxy resins with varying monomer molecular weights to alter network density. Their study revealed that the fracture toughness of the pure resin is almost independent of network density. However, the toughness of rubber-toughened epoxy resins is highly dependent on the resin structure. Epoxy resins with lower crosslink densities exhibit greater ductility and consequently higher toughness.

#### 2.2.4. Interfacial Interaction and Compatibility

The interfacial interaction and compatibility between the epoxy resin matrix and rubber are crucial factors influencing the toughening effect and remain a significant area of current research. For rubber and resin matrices that are not fully compatible, the interaction at the interface directly affects the bonding strength and the size and distribution of rubber particles.

Qian et al. [34] systematically investigated the effects of various shell compositions of core–shell particles—such as PMMA, P[MMA-acrylonitrile (AN)], [PMMA-glycidyl methacrylate (GMA)], and [PMMA-divinylbenzene (DVB)]—on the particle–epoxy resin interface. Their study demonstrated that the dispersion morphology of particles in the epoxy resin matrix plays a critical role in toughening the resin. This dispersion can be influenced by incorporating AN and GMA copolymer monomers into the PMMA shell or by crosslinking the shell. They also found that nanoscale interfacial interactions do not directly affect fracture toughness; instead, these interactions are used to control the morphology of the blend, which has a significant impact on toughness. Subsequent studies confirmed that the dispersion degree of particles is crucial for the fracture toughness of the modified epoxy resin, with a homogeneous particle distribution significantly enhancing toughness compared to microclustered forms [35].

### 2.3. Research Progress in Rubber Toughening Methods

The method of using rubber particles to toughen epoxy resin was first proposed by McGarry [36] in 1970, marking the inception of toughening epoxy resins using rubber. Currently, there are two main approaches for rubber toughening of epoxy resins: (1) liquid rubber toughening; (2) core–shell rubber particle toughening.

#### 2.3.1. Liquid Rubber Toughening Method

Liquid rubber, including types such as nitrile rubber, polybutadiene, and polysulfide rubber, is commonly used in toughening epoxy resins. Among these, liquid nitrile rubber systems are the most developed [28]. To enhance the interface interaction between the rubber and the resin matrix, active functional groups that can participate in the curing reaction are typically introduced into the rubber, such as carboxyl, hydroxyl, and amino groups [37].

Liquid rubbers with active reactive groups are miscible with epoxy resins before curing. During the curing process, these rubbers undergo reaction-induced phase separation, forming a two-phase structure (sea-island morphology). Ultimately, the rubber is dispersed as micron-sized particles within the epoxy resin matrix.

**Figure 2:**
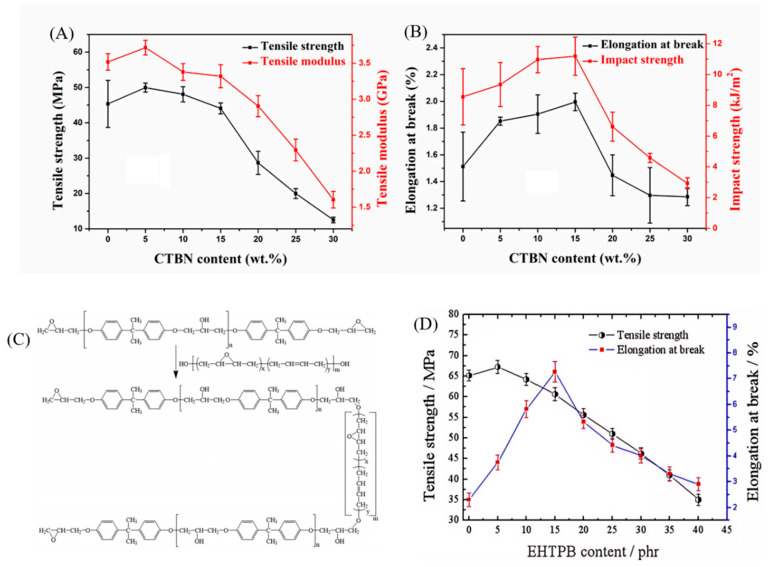
Effect of various CTBN contents on the mechanical properties of composite materials: (**A**) tensile strength and tensile modulus; (**B**) elongation at break and impact strength. Reproduced from Ref. [38] with permission of MDPI. (**C**) OH group chemistry diagram of EP group and EHTPB; (**D**) tensile strength and fracture elongation. Reproduced from Ref. [40] with permission of John Wiley and Sons.

Bian et al. [38] used carboxyl-terminated butadiene–nitrile liquid rubber (CTBN) to toughen boron nitride–epoxy resin hybrid systems and investigated the impact of varying CTBN content on the mechanical properties of the composites. As shown in Figure 2A, the tensile strength reached its maximum value of 50 MPa at a CTBN content of 5%. However, as the CTBN content continued to increase, the tensile strength gradually decreased, likely due to the aggregation of CTBN within the resin matrix, which prevented the formation of a well-dispersed structure. The elongation at break increased with higher CTBN content, reaching a maximum of 32% greater than the unmodified epoxy resin when CTBN content was 15%. At a CTBN content of 20%, the elongation at break decreased significantly, and the changes in impact strength paralleled those of the elongation at break (Figure 2B). The results indicate that the mechanical properties of the composites are optimal with CTBN content ranging from 5% to 15%.

Liu et al. [39] employed carboxyl-terminated nitrile rubber (CTBN) to toughen high-molecular-weight solid epoxy resins. When the CTBN content reached 10 wt%, the tensile strength increased to 105.4 MPa, the impact strength rose from 7.63 kJ/m^2^ to 23.9 kJ/m^2^, and the elongation at break improved from 5.4% to 8.1%. These improvements in impact strength and other properties significantly expanded the application range of the modified epoxy resin.

In addition to using liquid nitrile rubber, polybutadiene is also frequently used to toughen epoxy resins. Zhou et al. [40] utilized non-polar epoxy-terminated hydroxyl polybutadiene (EHTPB) to toughen epoxy resins (EP), aiming to enhance both impact strength and dielectric performance. EHTPB, containing hydroxyl and epoxy groups, readily undergoes chemical reactions with EP, thereby improving interface interactions and compatibility (Figure 2C). The results demonstrated that the tensile strength reached its maximum at 5 phr EHTPB, after which it gradually decreased. This decrease is attributed to the lower mechanical strength of EHTPB compared to EP, as EHTPB is a soft elastomer. Due to good chemical bonding between EHTPB and EP, the elongation at break significantly increased, peaking at 15 phr EHTPB before starting to decline (Figure 2D).

End-amine liquid nitrile rubber (ATBN), due to its active amino (-NH_2_) groups capable of participating in chemical reactions, is widely used for toughening and modifying epoxy resins. N. Chikhi et al. [30] incorporated varying amounts of ATBN, a copolymer containing 16% acrylonitrile and end-amine butadiene–acrylonitrile, into bisphenol-A-type epoxy resin cured with multi-amine imidazoline to enhance its toughness. The study revealed that the incorporation of ATBN resulted in a reduction in tensile strength and an increase in elongation at break, with a yield phenomenon observed. As the ATBN content increased, the tensile modulus decreased slightly from approximately 1.85 GPa to 1.34 GPa. The addition of 12.5 phr ATBN resulted in a threefold increase in impact strength, reaching 2.86 and 14.26 kJ/m^2^ for notched and unnotched samples, respectively. Additionally, the fracture toughness increased from 0.91 MPa·m^1/2^ to 1.49 MPa·m^1/2^, representing a 1.5-fold enhancement.

Dou et al. [9] investigated the impact of adjusting the acrylonitrile content in amine-terminated butadiene nitrile rubber (ATBN) on the phase separation of ATBN rubber-toughened epoxy resin networks. The study found that the particle size of the rubber is highly dependent on the acrylonitrile content in ATBN, which significantly affects the microstructure of the ATBN-toughened epoxy resin network. The phase separation scale is a crucial factor influencing network performance. Networks with larger phase separation scales exhibit longer elongation at break, while networks with smaller phase separation scales are more effective in improving impact strength.

The addition of rubber to epoxy resins for toughening can also lead to a reduction in mechanical properties such as tensile strength and flexural strength. To mitigate the impact on mechanical performance, nanoparticles can be introduced into the rubber/epoxy (EP) system to enhance its properties. Wang et al. [41] incorporated carboxyl-terminated butadiene–acrylonitrile (CTBN) into epoxy resin to improve fracture toughness, and then introduced graphene nanoplatelets (GnP) with two different lateral sizes (nominal diameters <1 µm (GnP-C750) and 5 µm (GnP-5)) into the CTBN/epoxy system to prepare multi-phase composites. Although the tensile strength and flexural strength of the CTBN/EP system are significantly lower compared to pure EP, the mechanical strength of the resin system improves with the addition of 1 wt% and 3 wt% GnP-C750. The inclusion of CTBN markedly enhances the fracture toughness of the epoxy resin, increasing it by approximately 70.7% compared to pure EP. When subjected to damage, cracks in the GnP/CTBN/EP composite system not only interact with CTBN but are also impeded by GnP, altering the crack path and thereby further enhancing the toughness of the epoxy resin composites.

#### 2.3.2. Core–Shell Rubber Toughening Method

The core–shell rubber toughening method has emerged as a rapidly developing approach for enhancing epoxy resins, as it addresses some limitations of liquid rubber, such as the uncontrolled particle shape, size, and distribution during reaction-induced phase separation. In core–shell rubber systems, the core is typically composed of rubber particles, while the shell can consist of one or more organic or inorganic materials. This core–shell structure allows for a synergistic effect between the two components, leveraging their individual properties [42].

The shell is often made from materials that exhibit good compatibility with the epoxy resin matrix, such as polymethyl methacrylate (PMMA). The core materials generally include substances like polybutadiene, acrylate–polyurethane, or siloxanes [43]. Under external forces, core–shell rubber particles undergo cavitation, leading to shear yielding of the matrix. The rubber particles at the crack tip are stretched to match the matrix’s dimensions. The deformation and failure of the rubber particles absorb energy, effectively impeding crack propagation [44].

**Figure 3:**
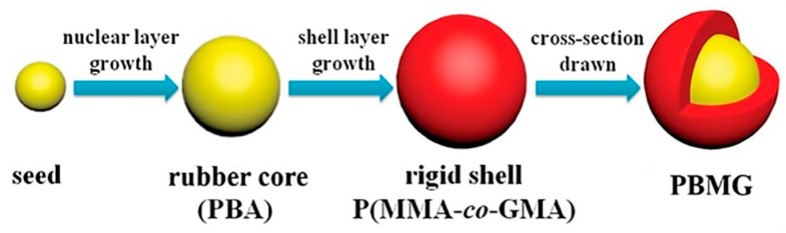
Schematic preparation of PBMG. Reproduced from Ref. [45] with permission of Royal Society of Chemistry.

Xu et al. [45] employed a seed emulsion polymerization method to synthesize a core–shell structured emulsion composed of poly(butyl acrylate)/poly(methyl methacrylate-methyl methacrylate glycidyl ester) (PBMG), which was then used to toughen epoxy resin (EP). The synthesis process of PBMG is illustrated in Figure 3. Their study revealed that both the impact strength and flexural strength of the EP/PBMG composite increased with the PBMG content. When the mass fraction of PBMG reached 5 wt%, it was uniformly dispersed in the EP matrix, achieving maximum impact strength and flexural strength of 37.25 kJ/m^2^ and 168.4 MPa, respectively.

**Figure 4:**
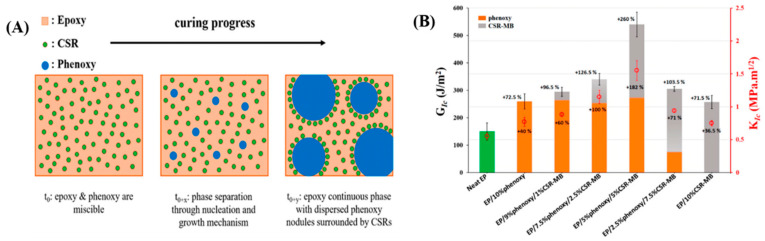
(**A**) Schematic representation of the morphologies of epoxy modified with phenoxy and CSR during curing. (**B**) Critical strain energy release rate (GIC) and corresponding critical stress intensity factor (KIC) of neat EP and toughened with 10 wt% phenoxy, phenoxy/CSR-MB (9/1, 7.5/2.5, 5/5, 2.5/7.5 wt%), and 10 wt% CSR-MB. Reproduced from Ref. [46] with permission of MDPI.

Velthem et al. [46] utilized phenoxy thermoplastic rubber and core–shell rubber (CSR) particles (polybutadiene and polymethyl methacrylate) for the simultaneous modification of epoxy resin. As shown in Figure 4A, prior to curing, the epoxy resin and phenoxy thermoplastic resin are miscible, forming a homogeneous blend, while CSR particles remain insoluble throughout the curing process and ultimately aggregate around the phenoxy phase. As depicted in Figure 4B, compared to pure epoxy resin, the addition of either phenoxy or CSR-MB alone increases the critical strain energy release rate (GIC) by approximately 73%. When both toughening agents are combined, particularly with a 5/5 wt% ratio of phenoxy and CSR-MB, the critical fracture energy increases by approximately 260%, and the critical stress intensity factor (KIC) value improves by about 182%.

Wang et al. [47] introduced a crosslinking agent, methyl methacrylate (ALMA), into the shell and core layers of core–shell particles (CSPs) and grafted epoxy-containing glycidyl methacrylate (GMA) onto the PMMA shell. They found that the toughening effect of the grafted GMA on CSP was superior to that achieved with the crosslinking agent alone. Compared to pure epoxy resin (EP), the KIC increased by 245%, from 0.85 MPa·m^1/2^ to 2.93 MPa·m^1/2^, and the impact strength improved by 87.5%, from 7.2 kJ/m^2^ to 13.5 kJ/m^2^.

To simultaneously enhance the mechanical properties and fracture toughness of epoxy resin without compromising heat resistance, Wang et al. [48] selected commercial triblock copolymers (PMMA-b-poly(butyl acrylate)-b-PMMA) and core–shell particles (core: polybutadiene, shell: PMMA) for modifying the epoxy resin system. They successfully prepared a hybrid modified epoxy resin system using a two-step processing method. The study demonstrated that the KIC of the block copolymer particle (BCP)/core–shell particle (CSP)/EP system (with 3 phr BCP and 5 phr CSP) increased by 91.14% compared to pure epoxy resin and by 11.5% and 21.2% compared to BCP/EP and CSP/EP systems, respectively. The flexural strength also showed significant improvements compared to pure epoxy, BCP/EP, and CSP/EP systems. It was the elongation of the rubber phase and the destruction of the BCP connections between the CSP and the epoxy resin that significantly increased energy absorption.

### 2.4. Summary

Rubber-toughened epoxy resins can significantly enhance the toughness and impact resistance of epoxy resins while offering good processability and reduced production costs. Through extensive research and development, rubber-toughened epoxy resins have demonstrated significant application value in various fields. However, there are notable drawbacks: rubber toughening can lead to decreased thermal resistance, reduced aging performance, and diminished thermal stability of the epoxy resin. Addressing these issues remains a pressing challenge.

## 3. Thermoplastic Resin Toughening Method

Since the 1980s, the use of thermoplastic resins to toughen epoxy resins has emerged as a new focus area. Thermoplastic resins offer several advantages, including good rigidity, high ductility, excellent toughness, high modulus, and superior thermal resistance [49]. When incorporated into an epoxy resin matrix, thermoplastic resins enhance the toughness of the epoxy without affecting, and in some cases even improving, its modulus and thermal resistance—benefits that traditional rubber toughening methods cannot achieve [50,51].

### 3.1. Reaction-Induced Phase Separation Theory

The comprehensive performance of epoxy resin/thermoplastic resin blend systems is closely related to the phase structure of the blend systems [52]. Homogeneous systems cannot significantly improve the fracture toughness of materials, while the construction of multi-phase morphology through dynamic phase separation during the curing process is the key to achieving toughening and strengthening [53].

In the late 1980s, Inoue et al. [54] introduced the concept of reaction-induced phase separation. Before or early in the curing reaction, when thermoplastic resins and epoxy resins are mixed, a homogeneous system is formed. At this stage, due to the small size of the chemical structures and molecules, the compatibility between the two is relatively good. As the curing reaction progresses, the molecular weight of the thermosetting resin gradually increases, reducing compatibility with the thermoplastic resin. This results in a thermodynamically incompatible system where phase separation begins, leading to the formation of a biphasic structure.

Pascault et al. [55,56] also investigated phase separation in thermoplastic resin/epoxy systems. They found that the development of phase structures is crucial for toughening epoxy resins, with double-continuous and phase-inverted structures providing more pronounced toughening effects. Li et al. [57,58] further studied the phase separation process in epoxy resins, proposing that, as the content of thermoplastic resins increases, the phase structure of the blend transitions from an island structure to a double-continuous structure and ultimately to a phase-inverted structure. The double-continuous phase structure provides the best toughness.

### 3.2. Thermoplastic Resin Toughening Mechanism

The toughening of epoxy resin with thermoplastic resins primarily involves mechanisms such as the crack pinning mechanism and bridging constraint effect. The crack energy dissipation mechanisms include crack deflection, crack bridging, hard particle debonding, interparticle rupture, shear band formation in the matrix, and void growth [59].

In the 1970s, Lange et al. [60] first proposed the crack pinning mechanism. This mechanism suggests that, under stress, as a crack tip with unit length propagates through the cured material, it encounters a series of curing particles tightly bonded to the matrix. At these particles, the crack path deviates, creating secondary cracks. In the initial stage, the formation of secondary cracks causes the crack front to exhibit non-linear characteristics, and this new crack front can absorb and store energy.

Based on Lange’s crack pinning mechanism, Sun et al. [61] proposed a bridge–crack pinning theory, which is well-suited for describing toughening in epoxy resins with thermoplastic resins. Thermoplastic resins often possess an elastic modulus comparable to that of epoxy resins and a much higher elongation at break than the matrix. This allows their particles to bridge across cracks in the brittle epoxy resin, constraining and closing the cracks. The bridging effect of thermoplastic resin particles not only restrains the overall propagation of the crack front but also acts as a pinning force on the crack, resulting in a wavy, bow-shaped crack front.

### 3.3. Influencing Factors

The molecular structure, molecular weight, and inherent properties of thermoplastic resins significantly impact the microstructure and overall performance of the blended system. Additionally, factors such as the dispersion of thermoplastic particles within the matrix, particle size, the mechanical strength of the particles themselves, the adhesion between the particles and the matrix resin, and the uniformity of particle distribution within the resin matrix are crucial determinants of the toughening effect in multi-phase systems [62].

### 3.4. Advancements in Thermoplastic Resin Toughening

In 1983, Bucknall and Partridge [63] introduced the concept of using thermoplastic resins to modify and toughen epoxy resins. Currently, the thermoplastic resins employed for toughening epoxy resins include polyetheretherketone (PEEK), polyetherimide (PEI), polysulfone (PSF), and polyethersulfone (PES). These materials are favored not only for their excellent toughness but also for their high modulus and thermal resistance, making them well-suited as toughening agents for epoxy resins.

#### 3.4.1. Polyphenylene Sulfone

Polyphenylene sulfone (PSF) is an amorphous, transparent polymer characterized by its high strength and exceptional thermal stability. It exhibits excellent mechanical properties and dimensional stability. Furthermore, the structural similarity between PSF and bisphenol-A-type epoxy resins results in good compatibility between the two materials.

Zheng et al. [64] incorporated carboxylated carbon nanotubes (CNT-COOHs) into an epoxy resin (EP)/polyphenylene sulfide (PSF) blend to control the phase separation behavior of the ternary composite materials. The study demonstrated that the addition of CNT-COOHs enhanced the complex viscosity and curing reaction rate of the EP/PSF blend, significantly suppressing the early termination of the phase separation process. Additionally, the presence of CNT-COOHs improved the fracture toughness, mechanical properties, and thermal performance of the EP/PSF/CNT-COOH composites.

#### 3.4.2. Polyether Sulfone

Polyether sulfone (PES) is one of the earliest thermoplastic resins used to toughen epoxy resins, known for its excellent thermal stability and chemical resistance. Mimura’s research on PES-toughened epoxy resins revealed that the toughest results are achieved when the modified system exhibits a cocontinuous phase structure [65].

Jiang et al. [66] utilized hydroxyl-terminated polyether sulfone as a toughening agent and employed a blending method to modify a single-component high-performance epoxy resin. The study indicated that the toughening of the blend is primarily achieved through bridging effects and crack pinning effects. As the PES content increased, there was a significant improvement in both the fracture toughness and impact strength of the samples compared to pure epoxy resin. When the PES content reached 15 parts, the fracture toughness of the blend increased to 2.90 MPa·m^1/2^, a 32% improvement over the unmodified resin; simultaneously, the impact strength rose to 83.7 J/m.

**Figure 5:**
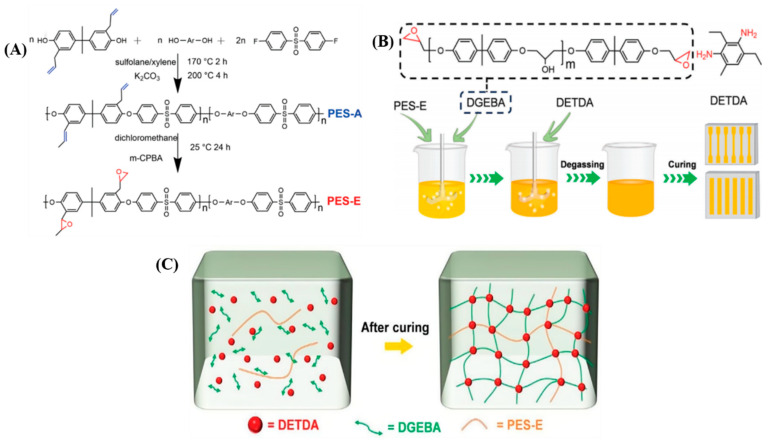
(**A**) Synthesis process of PES-E; (**B**) synthesis of PES-E/DGEBA composite; (**C**) schematic illustration of crosslink network formation of PES-E/DGEBA composite. Reproduced from Ref. [65] with permission of John Wiley and Sons.

To enhance the toughening effect, Xu et al. [67] synthesized epoxy-functionalized polyether sulfone (PES-E) (Figure 5A), which can participate in the curing reaction of epoxy resins. PES-E was mixed into bisphenol A diglycidyl ether (DGEBA) at weight ratios of 5%, 10%, 15%, and 20% and then cured with diethyltoluenediamine (DETDA) (Figure 5B). The study found that, at 5% PES-E content, the tensile strength, elongation at break, and impact strength of the PES-E/DGEBA composites were maximized, showing increases of 5.5%, 21.9%, and 74.1%, respectively, compared to pure epoxy resin. As depicted in Figure 5C, PES-E not only dispersed uniformly within the epoxy resin matrix but also crosslinked with the epoxy resin through cocuring reactions, forming a crosslinked semi-interpenetrating polymer network that bears stress along with the epoxy molecular chains.

In traditional methods, polyether sulfone (PES) is often used in blended forms to toughen epoxy resins. In addition to blending, PES fibers have also been utilized to enhance the toughness of epoxy resins. Cheng et al. [68] designed and prepared a uniformly oriented PES fiber mat as an interleaved layer to simultaneously improve both the Mode I and Mode II fracture toughness of carbon fiber/epoxy composites. The study revealed that, compared to laminates without the interleaved layer, the introduction of a 28.3 g/m^2^ PES fiber mat increased the Mode I and Mode II fracture toughness by up to 120% and 68.8%, respectively. Additionally, the interleaved layer improved the interlaminar shear strength and compressive after-impact performance by 18.2% and 43.8%, respectively.

#### 3.4.3. Polyimide

Polyimide (PI) is a high-performance polymer known for its exceptional thermal stability and mechanical properties, making it suitable for applications in aerospace, automotive manufacturing, and microelectronics [69,70,71]. Extensive research indicates that PI can be an effective additive for enhancing the mechanical and thermal properties of epoxy resins [72,73,74].

Ji et al. [75] synthesized a novel polyimide with trifluoromethyl groups (PIS) and used it to improve the toughness of epoxy resins. When the polyimide content was 7.5 wt%, the tensile strength reached a maximum value of 92.3 MPa, an increase of approximately 128% compared to the unmodified epoxy resin. The fracture toughness increased from 0.86 MPa·m^1/2^ to 1.56 MPa·m^1/2^, representing an 81.4% improvement.

Polyether imide (PEI), a type of polyimide, exhibits similar excellent properties. Chen et al. [11] investigated the use of low-content PEI in conjunction with amino-functionalized multi-walled carbon nanotubes (NH_2_-MWCNTs) to toughen epoxy resins. They found that, when the amount of NH_2_-MWCNTs was 0.4 parts and PEI was 5 parts, the KIC reached its maximum value, showing a 73.1% increase compared to pure epoxy resin, a 29.8% increase compared to PEI/EP binary composites, and a 45.1% increase compared to NH_2_-MWCNTs/EP binary composites. In this composition, the flexural strength of the composite also reached its peak at 145.7 MPa, which is 26.8% higher than that of PEI/EP composites and 41.4% higher than that of NH_2_-MWCNTs/EP composites. Additionally, during the reaction-induced phase separation process, the distribution of carbon nanotubes was observed to preferentially associate with PEI microspheres, which is likely related to the significant enhancement in toughness.

### 3.5. Summary

Toughening epoxy resins with thermoplastic resins not only enhances toughness but also maintains their modulus and thermal resistance [75]. However, there are drawbacks: thermoplastic resins have poor solubility and flowability, and their use in modifying epoxy resins often requires large quantities, which increases the viscosity of the epoxy system and worsens processability. As technology continues to advance, this field is expected to see more innovations and breakthroughs.

## 4. Hyperbranched Polymer Toughening

Hyperbranched polymers (HBPs) are a class of highly branched three-dimensional macromolecules characterized by numerous branching points, which prevent extensive chain entanglement. They feature a high density of terminal functional groups, leading to increased reactivity, reduced viscosity, and higher solubility. These properties facilitate modifications and functionalization, making HBPs suitable for synthesizing a variety of functional materials [76,77]. As the demand for improved performance in polymer composites grows, researchers have begun to explore the incorporation of hyperbranched polymers into epoxy resins and other resin systems to enhance the materials’ toughness, strength, and impact resistance.

### 4.1. Toughening Mechanism

When hyperbranched polymers (HBPs) are used as non-reactive modifiers, phase separation occurs, and the toughening mechanism resembles that of rubber toughening. For cases where phase separation does not occur, researchers have conducted in-depth studies. Liu et al. [5] synthesized terminal epoxy hyperbranched aromatic polyethers (EHBPEs) to toughen epoxy resins. They found that adding 5% EHBPE optimized the material performance. They proposed a toughening mechanism wherein the introduction of HBPs expands the material’s free volume and enhances the mobility of the molecular chains. Additionally, the nearly spherical structure of HBPs effectively absorbs impact energy from various directions, thereby significantly improving the resin’s toughness and impact strength.

Zhang et al. [76] conducted an in-depth study on the modification of epoxy resins with hyperbranched polymers (HBPs) and proposed an in situ uniform reinforcement and toughening mechanism. In this mechanism, the terminal groups of HBPs participate in the curing reaction of the epoxy matrix, while HBPs can disperse and permeate into the molecular chains of the resin matrix. This interaction increases the molecular chain interactions during the curing process, ultimately enhancing the tensile and flexural strengths. During impact, numerous non-crosslinked molecular voids absorb energy, deform, and form fibrils, exhibiting high toughness. Consequently, HBPs provide a dual effect on epoxy resins by both reinforcing and toughening them.

### 4.2. Current Research

Current research indicates that significant progress has been made in using hyperbranched polymers to toughen epoxy resins. Researchers are focusing on optimizing the toughening effect by adjusting the structural design of hyperbranched polymers, such as core–shell structures and branched architectures. This includes incorporating terminal groups that can react with the epoxy resin to enhance dispersion and compatibility within the resin matrix. These advancements aim to improve the toughening effect and achieve multi-faceted control over the material properties of epoxy resins, thereby meeting the diverse needs of different application fields [5,78].

#### 4.2.1. Hyperbranched Polyesters (HBPEs)

Currently, there are intensive studies on hyperbranched polyesters, with HBPE being a highly branched polymer known for its unique branching morphology and increased free volume. These characteristics make HBPE widely applicable in enhancing material flexibility and toughness, as well as in nanotechnology and material modification [79]. HBPE primarily includes hydroxyl-terminated hyperbranched polyesters and epoxy-terminated hyperbranched polyesters, with hydroxyl-terminated variants being more thoroughly studied [80].

In 1999, Boogh et al. [81] were the first to use hyperbranched polymers to toughen epoxy resins. Their research showed that adding just 5% HBPE to the epoxy system increased the critical strain energy release rate to 720 J/m^2^, which is approximately six times higher than that of the unmodified resin. Additionally, the KIC improved by about 2.5 times. Importantly, the glass transition temperature (T_g_), elastic modulus, and processing performance of the modified epoxy resin were comparable to those of the unmodified epoxy resin, a feat not achieved by previous toughening agents.

Yang et al. [82] used hydroxyl-functionalized hyperbranched polymer (H30) to improve the mechanical properties of DGEBA epoxy resin at liquid nitrogen temperatures (77 K). The terminal hydroxyl groups in H30 can react with an anhydride, covalently bonding with the epoxy matrix, while unreacted hydroxyl groups form numerous hydrogen bonds. When the H30 content was 10%, the modified epoxy resin system achieved a tensile strength of 115.6 MPa, a 17.7% increase over pure epoxy resin, and the impact strength improved by 26.3%.

Fei et al. [83] synthesized a series of end-carboxyl hyperbranched polyesters (HBPE-COOHs) with various main chain structures using a simple A2 + B3 one-step method. These HBPE-COOH polymers were introduced into an epoxy/anhydride curing system. The study demonstrated that the terminal carboxyl groups of HBPE-COOH facilitate the curing process of the epoxy/anhydride system, leading to simultaneous improvements in elongation at break and tensile strength. The epoxy resin with 5% HBPE-COOH2 exhibited a maximum impact strength of 36.4 kJ/m^2^, which is a 178% increase compared to pure epoxy resin. Additionally, the epoxy resin with 3% HBPE-COOH3 achieved a fracture elongation of 5.25%, significantly enhancing toughness.

#### 4.2.2. Terminal Epoxy Group Hyperbranched Polymers

In 1996, Sorensen et al. [84] first introduced hyperbranched polymers with terminal epoxy groups. The epoxy end groups exhibit good compatibility with epoxy resin systems, and by adjusting the content of these epoxy end groups, the phase separation process and mechanical properties of the modified epoxy resins can be controlled.

Varley et al. [85] used an epoxy-functionalized hyperbranched polymer (HBP) as an additive in an epoxy–anhydride resin system. Their research demonstrated that, within the additive concentration range of 0 to 20 wt%, HBP significantly increased the fracture toughness of the epoxy resin, with minimal adverse effects on the processing and durability of the cured resin system. However, the addition of HBP resulted in approximately a 30% reduction in both the flexural modulus and stress, and a decrease of about 10% in the glass transition temperature (Tg).

Zhu et al. [86] synthesized a novel terminal epoxy hyperbranched polyether sulfone (EHPES) using a one-step method. They used EHPES to modify bisphenol A diglycidyl ether (DGEBA). Their study found that the copolymer containing 7% EHPES achieved tensile strength, flexural strength, and impact strength of 87.75 MPa, 112 MPa, and 41.70 kJ/m^2^, respectively, showing improvements of 9.24%, 10.70%, and 19.38% over unmodified DGEBA.

#### 4.2.3. Hyperbranched Polysiloxanes

Hyperbranched polysiloxanes (HBPSs) are a type of polymer material characterized by a three-dimensional network structure composed of silicon, oxygen, and organic groups, synthesized through specific polymerization reactions [87]. This structure endows HBPS with unique physicochemical properties, such as excellent thermal stability, superior mechanical performance, and low surface energy. In the context of toughening epoxy resins, HBPS has garnered attention for its effective toughening capabilities [88].

Pan et al. [89] synthesized an epoxy-terminated hyperbranched polysiloxane (EPTS-12) and incorporated it as a toughening agent into epoxy resins. Both at room temperature and low temperatures, the tensile strength and elongation at break of the epoxy resin increased with the addition of EPTS-12, initially rising and then declining. When the EPTS-12 content was 5 wt%, the maximum tensile strengths were 89.55 MPa at room temperature and 116.68 MPa at −72 °C, while the maximum elongations at break were 14.19% and 16.06%, respectively. These values represent increases of 35.66% and 74.19% compared to the unmodified resin.

**Figure 6:**
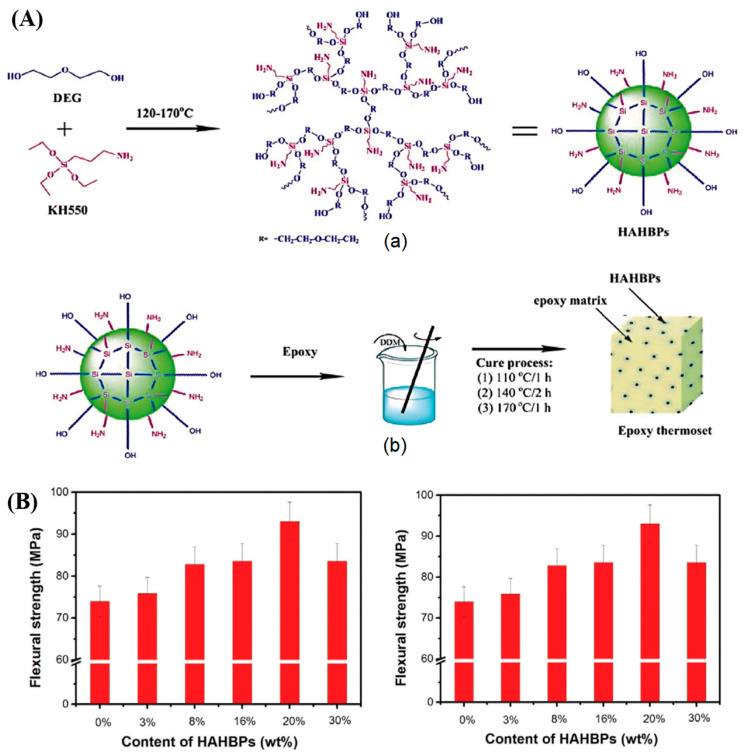
(**A**) (**a**) Synthesis of HAHBPs and (**b**) Schematic diagram of preparation process of HAHBP-modified E51 epoxy resin; (**B**) Effect of HAHBP dosage on bending strength and impact strength of E51-DDM/HAHBPs resin system. Reproduced from Ref. [90] with permission of John Wiley and Sons.

Chen et al. [90] utilized the A2 + B3 “one-pot” method to synthesize a novel silicon-containing hyperbranched polysiloxane (HAHBP) for the modification of epoxy resins (Figure 6A). The study demonstrated that, compared to unmodified resins, the addition of HAHBPs significantly improved the mechanical properties of the epoxy resin. Specifically, as shown in Figure 6B, incorporating 3%, 8%, 16%, 20%, and 30% HAHBPs resulted in increases of 2.6%, 12.0%, 13.0%, 25.7%, and 13.0% in flexural strength, respectively. Additionally, the impact strength of the epoxy resin increased progressively with the amount of HAHBPs added, with an 8% HAHBP content improving the impact strength by 70%.

### 4.3. Summary

Hyperbranched polymers (HBPs) enhance epoxy resins by improving toughness, reducing viscosity, and increasing processability without compromising thermal resistance. Due to their compact structure, HBPs have lower viscosity compared to thermoplastic resins, making them suitable for low-viscosity epoxy resin composite manufacturing technologies. However, the complex synthesis and high cost of HBPs limit their widespread application in the toughening of epoxy resins. Therefore, developing simple and efficient synthesis methods for HBPs is a key focus for future research.

## 5. Flexible Chain Segment Toughening Method

Flexible chain segments are components within polymeric materials characterized by their flexible and bendable molecular structures. These segments typically include groups such as C—O, C=C, C—Si, C—N, and Si—O. The molecular structure and properties of these segments impart greater flexibility and deformability to the entire polymer.

Incorporating flexible chain segments into polymer materials can enhance their elasticity, toughness, and processability. This modification improves the material’s ability to absorb and dissipate energy, making it more suitable for a variety of applications. The introduction of flexible chain segments allows for better performance in demanding conditions by improving the overall mechanical properties and expanding the potential uses of the material in various fields.

### 5.1. Toughening Mechanism

Flexible chain segments can create dispersed stress concentration points within the material. When subjected to external forces, these segments absorb and dissipate energy through chain extension and rotation, thereby enhancing the material’s toughness [91]. The introduction of flexible chain segments also helps reduce volumetric shrinkage during the curing process of the epoxy resin, thereby lowering internal stresses and preventing the formation and propagation of cracks. Furthermore, the compatibility of flexible chain segments with the epoxy resin matrix improves interfacial bonding strength, further enhancing the overall performance of the material.

### 5.2. Current Research

Toughening epoxy resins with flexible chain segments involves incorporating various flexible chains into the cured system through molecular design strategies, either by modifying the curing agent or directly altering the epoxy resin. There are two primary methods for this approach [92]: (1) incorporating flexible chain segments into the curing agent; (2) directly introducing flexible chain segments into the epoxy resin.

#### 5.2.1. Toughening Epoxy Resins with Flexible Chain Segments in Curing Agents

Flexible curing agents, due to the free rotation of their molecular chain segments, exhibit high flexibility and adaptability. During the curing process, these flexible chains bond to the dense crosslinked network of the epoxy resin, forming a more relaxed crosslinked structure [91]. Under applied stress, the molecular chains have more room to move, absorbing impact energy through deformation and reorientation of the segments, which effectively prevents crack propagation [92,93].

**Figure 7:**
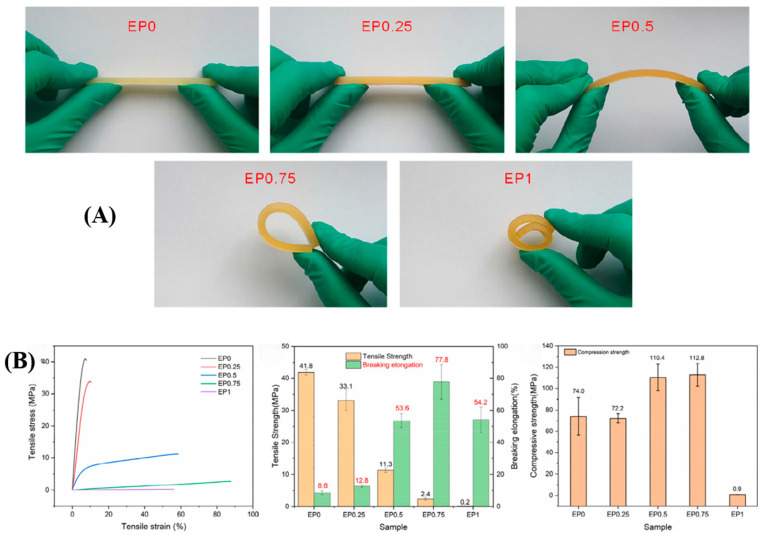
(**A**) Photographs of EP0, EP0.25, EP0.5, EP0.75, and EP1 bending test; (**B**) tensile stress–strain curve, tensile test results, and compression test results. Reproduced from Ref. [19] with permission of John Wiley and Sons.

Dai et al. [19] employed a thiol-ene click reaction to introduce flexible chain segments into a polymer network, thus toughening a flexible and hydrophobic epoxy resin (DGEBDBP). The study found that the flexibility of the epoxy resin increased with the addition of DGEBDBP (Figure 7A). When the mass fractions of DGEBDBP and E44 were 75% and 25%, respectively (EP0.75), the composite achieved the highest elongation at break, reaching 77.8%, which is nine times that of pure epoxy resin. Additionally, the compressive strength also peaked at 112.8 MPa (Figure 7B).

Wang et al. [91] synthesized a novel amine with flexible poly(propylene oxide) side chains (AFPE) as a curing agent for bisphenol A diglycidyl ether (DGEBA), resulting in a high-toughness epoxy resin. The study showed that the epoxy resin modified with AFPE 40-C achieved a fracture elongation of 22.3%, which is an increase of 150.5% compared to pure epoxy resin, and the impact resistance improved by 46.2%.

#### 5.2.2. Incorporation of Flexible Chains into Epoxy Resins

Through molecular design strategies, traditional rigid chains can be replaced with flexible chains, fundamentally enhancing the material’s toughness. The core of this design approach lies in minimizing or avoiding the use of rigid structures, instead employing more flexible molecular architectures. By adopting non-cyclic or low-cyclic molecular structures, the intermolecular interactions can be effectively reduced, thereby increasing the overall flexibility of the material.

Lin et al. [94] conducted a study where polydimethylsiloxane (PDMS) oligomers were reacted with bisphenol A epoxy resin to prepare a silicone-modified epoxy resin (ESDG) copolymer. The results demonstrated that, when the PDMS content was 10%, the impact strength of the modified epoxy resin reached 22.0 J/m, representing a 115% increase compared to the unmodified resin. When the PDMS content was increased to 40%, the impact strength further increased to 59.0 J/m, a 478% improvement.

The addition of reactive diluents to epoxy resins introduces flexible chain segments while reducing viscosity and enhancing mechanical properties. Liu et al. [95] synthesized a low-viscosity reactive modifier, diethylene glycol diglycidyl ether (DGEG), and incorporated it into epoxy resin (EP) via a solvent-free method. The mechanical properties of the modified epoxy resin were investigated through tensile and impact tests. The results showed that the elongation at break and impact strength of EP significantly increased with higher DGEG content, while an optimal DGEG content also enhanced the tensile strength of EP.

Yang et al. [96] synthesized and characterized two novel epoxy resins—diglycidyl ether of ethoxylated bisphenol A (BPA) with two and six oxyethylene units (DGEBAEO-2 and DGEBAEO-6). DGEBAEO-6 was employed to toughen the conventional epoxy resin diglycidyl ether of BPA (DGEBA), and the thermal and mechanical properties of the modified system were systematically investigated. As the DGEBAEO content increased, the modified resin exhibited reduced viscosity, a lower glass transition temperature (Tg), and enhanced impact strength, while maintaining favorable thermal stability and mechanical performance. SEM analysis revealed that DGEBAEO-6, with elastomeric characteristics, effectively improved the toughness of DGEBA. The toughening mechanism is attributed to the flexible chain segments promoting plastic deformation within the epoxy network.

### 5.3. Summary

By introducing flexible segments, the reduction in rigidity and the increased molecular freedom of epoxy resins provide enhanced fracture toughness, allowing for more effective energy absorption. Additionally, the modified epoxy resins exhibit improved dispersion, reducing agglomeration and thereby enhancing the uniformity and performance stability of the material. As the technology for toughening epoxy resins with flexible segments matures, it is anticipated to find widespread applications across various fields.

## 6. Nanoparticle Toughening Method

Nanoparticle toughening, characterized by its synergistic effect of simultaneously reinforcing and toughening polymers, has emerged as a prominent research focus in materials science. Nanoparticles refer to ultrafine materials with particle sizes ranging from 1 to 100 nm, bridging the dimensional gap between atomic/molecular scales and macroscopic systems [97]. Owing to their large specific surface area, elevated surface atomic occupancy ratio, and enhanced chemical reactivity, nanoparticles can establish robust chemical bonding with epoxy resin matrices This interfacial interaction facilitates the formation of an ideal interface that induces crack blunting and hinders crack propagation, thereby achieving the goal of toughening [98,99].

### 6.1. Nano-SiO_2_

Kim et al. [100] utilized silane coupling agents to modify the surface of colloidal silica (12 nm) in a two-step process, resulting in a low-viscosity silica/epoxy hybrid resin. Their study demonstrated that, with 20% SiO_2_ addition, the tensile strength of the composite material reached 97.9 MPa, which is a 44% increase compared to pure epoxy resin. The elongation at break improved from 3.5% without SiO_2_ nanoparticles to 5.5–6.6%, and the impact strength of samples containing 3% SiO_2_ reached 15.8 kJ/m^2^, representing a 54.9% enhancement over pure epoxy resin. The interpenetrating network polymer (IPN) toughening method, which constructs crosslinked networks, increases the mutual support among molecular chains, enabling epoxy resin to more effectively dissipate and absorb energy under stress.

Pham et al. [101] prepared nano-SiO_2_ particles with a particle size distribution of 40–80 nm using rice husk as raw material and applied them to toughen epoxy resin (EP). The study demonstrated that, when the content of nano-SiO_2_ reached 0.07 parts in the epoxy resin, the tensile strength, modulus, and KIC of the epoxy resin increased by 7.23%, 4.37%, and 16.3%.

### 6.2. CNTs

Chen et al. [102] investigated the effects of incorporating toluene-functionalized carbon nanotubes (CNTs) into epoxy resin. The study demonstrated that the introduction of functionalized CNTs significantly enhanced the glass transition temperature (Tg), thermal conductivity, tensile strength, and fracture strength of the epoxy resin. Furthermore, the incorporation of functionalized CNTs resulted in improvements of 60%, 27%, and 80% in tensile, flexural, and fracture strengths, respectively.

Cha et al. [103] functionalized carbon nanotubes (CNTs) via ball milling and incorporated them into epoxy resin to fabricate high-performance nanocomposites. The study revealed that non-covalently modified CNTs (M-CNTs), compared to pristine CNTs, significantly enhanced the elastic modulus, tensile strength, and fracture toughness of the epoxy composites. Notably, the 2 wt% M-CNTs/epoxy composite exhibited a 64% increase in elastic modulus, a 22% improvement in tensile strength, and a remarkable 95% enhancement in fracture toughness.

### 6.3. Hybrid Epoxy Nanocomposite

In recent years, hybrid epoxy nanocomposites have emerged as a research focus in the polymer materials field due to their unique synergistic reinforcement mechanisms. By introducing nanoparticles (e.g., nano-SiO_2_, graphene oxide) into conventional modification systems (such as liquid rubber/epoxy resin or thermoplastic resin/epoxy resin), multi-scale reinforcement networks can be constructed, where the synergistic interaction between these components overcomes the limitations of single-modification mechanisms [104,105].

#### 6.3.1. Liquid Rubber/Epoxy Resin Nanocomposite

Raneesh Konnola et al. [10] successfully attached carboxyl-terminated poly(acrylonitrile–butadiene) copolymer (CTBN) to graphene oxide (GO) surfaces via anionic polymerization, forming a novel nanocomposite (GCTBN), which aims to enhance the composite performance. The results demonstrated that GCTBN exhibits excellent thermal stability and significantly improves the tensile strength, modulus, and fracture toughness of the composites, showing increases of 25%, 34%, and 128%, respectively, compared to neat epoxy resin.

Chen et al. [106] introduced potassium aluminum oxide (K-Al_2_O_3_) nanosheets and carboxyl-terminated butadiene–acrylonitrile (CTBN) liquid rubber into an epoxy resin system to fabricate novel K-Al_2_O_3_/CTBN/epoxy resin composites. They systematically investigated the effects of K-Al_2_O_3_ nanosheet content on the thermal and mechanical properties and morphology of the composites. The results indicated that the tensile modulus and elongation at break of the composites exhibited an initial increase followed by a decrease with increasing K-Al_2_O_3_ nanosheet content, reaching optimal values at 5 phr K-Al_2_O_3_ nanosheets. Specifically, these values increased by approximately 22% and 50%, respectively, compared to those of the pure epoxy resin. Additionally, the K-Al_2_O_3_ nanosheets effectively enhanced the thermal stability and glass transition temperature (Tg) of the composites.

Liu et al. [107] investigated the morphology and properties of epoxy resin composites synthesized via a high-pressure mixing method, incorporating organically modified montmorillonite (OMMT) and carboxyl-terminated butadiene–acrylonitrile (CTBN) rubber. Experimental results demonstrated that, compared to the pristine resin, the fracture toughness parameters (KIC and GIC) exhibited maximum increases of 2.2-fold and 7.6-fold, respectively, at loadings of 6 phr OMMT and 20 phr CTBN. A positive synergistic effect on fracture toughness was observed in the epoxy matrix modified with both rubber and OMMT. Additionally, the ultimate strength, yield strength, and glass transition temperature (Tg) of the OMMT/CTBN-modified epoxy resin were also enhanced.

#### 6.3.2. Core–Shell Rubber/Epoxy Resin Nanocomposites

Yang et al. [108] systematically investigated the regulation mechanisms of material properties by individually and synergistically incorporating core–shell rubber (CSR) and carboxyl-functionalized carbon nanotubes (CNTs) into a benzoxazine–epoxy–phenolic (BEP) ternary thermosetting resin system. The results demonstrated that CSR enhanced the toughness by 61% through cavitation effects and matrix shear yielding, while CNTs improved the flexural strength by 8% via bridging effects and interfacial reinforcement. The synergistic combination of CSR and CNTs further achieved a 160% increase in toughness and a 30% enhancement in strength, accompanied by an elevation of the glass transition temperature (Tg) from 193.6 °C to 196.8 °C, with the thermal decomposition temperature remaining stable at 360 °C. Microscopic analysis confirmed that the uniform dispersion of CSR induced plastic deformation, and the carboxyl groups on CNTs catalyzed curing while strengthening interfacial bonding. The performance optimization was attributed to the synergistic cavitation–bridging–pull-out multi-mechanisms enabled by CSR and CNTs.

#### 6.3.3. Thermoplastic Resin/Epoxy Resin Nanocomposite

Wang et al. [109] systematically investigated the synergistic toughening mechanisms of poly(sulfone) (PSF) and graphene oxide (GO) in a diglycidyl ether of bisphenol A (DGEBA) epoxy system. Experimental results demonstrated that the ternary composite containing 5 phr PSF and 0.2 phr GO exhibited a fracture toughness (KIC) of 1.88 MPa·m^0.5^, representing an 89.90% increase compared to the neat epoxy (0.99 MPa·m^0.5^), significantly surpassing the individual enhancements achieved by PSF (60.6%) or GO (79.8%) alone. Furthermore, the tensile strength increased by 8.80% (from 83.28 MPa to 90.61 MPa), the elongation at break improved by 53.10% (from 4.67% to 7.15%), and the Young’s modulus showed a slight enhancement. Thermal analysis revealed that the glass transition temperature (Tg) rose from 157.9 °C to 161.0 °C, while the thermal decomposition temperature remained stable at 360 °C. Microscopic characterization confirmed that PSF induced matrix plastic deformation through uniform dispersion, while GO enhanced interfacial bonding via bridging effects and crack pinning. The synergistic optimization of mechanical properties was attributed to the multi-mechanistic contributions of cavitation–bridging–pull-out interactions between PSF and GO.

Park et al. [110] investigated the dispersion of nanoclay particles in poly(methyl methacrylate) (PMMA) using epoxy resin as a dispersant. Through melt-mixing techniques, the dispersion state of nanoclay particles in PMMA at different ratios and their effects on the mechanical properties of the material were studied. Experimental results showed that, when the mass ratio of epoxy resin to clay was 10, a fully exfoliated state of clay particles with a nanoscale dispersion structure was achieved, significantly improving the tensile strength and impact toughness of the material.

Fereidoon et al. [111] investigated the mechanical properties of ternary composites fabricated by incorporating multi-walled carbon nanotubes (MWCNTs) and high-impact polystyrene (HIPS) into epoxy resin, aiming to enhance their comprehensive mechanical performance. The study demonstrated that the addition of MWCNTs and HIPS significantly improved the tensile strength, compressive strength, and impact strength of the composites by 52%, 43%, and 334%, respectively, while the flexural strength showed no notable enhancement. Additionally, the elongation at break of the composites also increased.

#### 6.3.4. Block Copolymer-Based Hybrid Epoxy Nanocomposites

Jayan et al. [112] incorporated graphene-oxide-grafted triblock copolymer (GO-g-TBCP, particle size range: 6–11 nm) into epoxy resin and analyzed the mechanical properties of the composite. The results demonstrated that, for the 0.5 wt% GO-g-TBCP composite, the tensile strength and KIC increased by 32.7% and 397.9%, respectively, compared to pure epoxy resin. Additionally, the glass transition temperature (Tg) rose from 168 °C to over 185 °C.

Gómez-del Río et al. [113] investigated the effects of incorporating a triblock copolymer (SBM) and carbon nanotubes (CNTs) into epoxy resin to enhance its fracture toughness. The results revealed that the composites exhibited significantly improved fracture toughness and energy absorption capabilities compared to the pure epoxy resin. Specifically, the addition of 5 phr SBM increased the fracture toughness by approximately 115%, while the composite containing only 0.25 phr CNTs showed a moderate enhancement of 14.5%. Interestingly, no significant synergistic effect was observed when SBM and CNTs were added simultaneously, which may be attributed to the increased plastic cavity size induced by CNTs, thereby diminishing the toughening contribution from SBM.

### 6.4. Summary

The advantages of nanoparticle-toughened epoxy resin lie in their large specific surface area and strong interfacial effects, which significantly enhance the fracture toughness and impact resistance of the material. Meanwhile, hybrid epoxy nanocomposites achieve further breakthroughs in performance through the synergistic combination of nanofillers and original modifiers. This collaboration optimizes the strength–toughness balance, providing innovative material solutions for aerospace, electronic packaging, and other fields, which integrate high mechanical performance with process feasibility.

## 7. Computational and Intelligent Method-Driven Development of Epoxy Resins

The performance optimization of epoxy resin systems has long relied on trial-and-error experimental research, which not only increases R&D costs but also faces inherent challenges in achieving precise reverse “structure–property” design. This limitation stems from the complex interplay of multi-component interactions and multi-scale property responses, rendering purely experimental approaches insufficient for establishing accurate structure–property relationships [114,115,116]. In recent years, parallel advancements in computational modeling techniques and machine learning methodologies have provided novel pathways for characterizing the physical properties of epoxy resins and predicting their performance [117,118,119].

### 7.1. Computational Modeling Techniques

Current advancements in the development and optimization of enhanced epoxy resin systems are increasingly benefiting from computational modeling techniques. Molecular dynamics (MD) simulations and finite-element-based multi-scale approaches have provided critical insights into phase behavior [120,121], curing kinetics [122,123], and fracture mechanics across multiple length and time scales [124,125,126].

**Figure 8:**
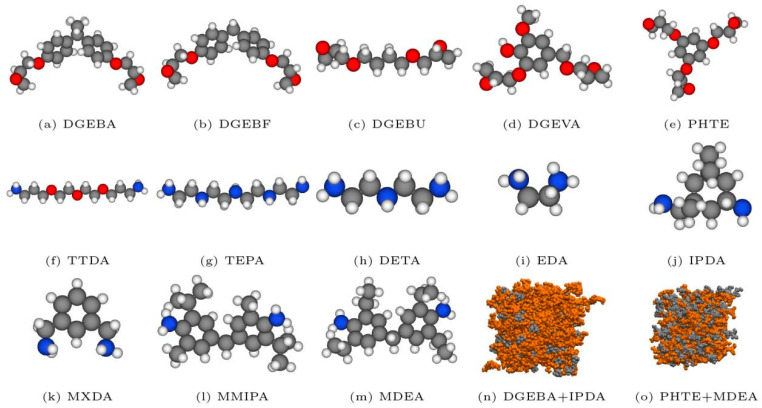
Molecular representation of the epoxy resins (**a**–**e**) and of the hardeners (**f**–**m**). Carbon, oxygen, nitrogen, and hydrogen atoms are shown in gray, red, blue, and white, respectively. (**n**,**o**) Snapshots of crosslinked systems, where orange and gray phases represent respectively the epoxy resins and hardeners [127].

Mathilde Orselly et al. [127] conducted a comprehensive investigation into the thermodynamic and mechanical properties of various epoxy–amine resins (as illustrated in Figure 8) using molecular dynamics (MD) simulations with the CHARMM force field. A multi-step crosslinking algorithm was first developed to simulate the resin curing process, followed by calculations of key material properties, including density and glass transition temperature. The results demonstrated that the simulation methodology effectively predicted these properties, with mean absolute deviations ranging from 2% to 12%. Furthermore, systematic analysis revealed the influence of molecular weight and crosslinking density on resin performance: increasing molecular weight and crosslinking density enhanced material density, while inversely affecting glass transition temperature and Young’s modulus. These findings not only validate the robustness of the adopted computational framework but also provide critical theoretical guidance for the rational design of high-performance epoxy–amine resin systems.

Zeng Lijian et al. [128] investigated the effects of uniform dispersion versus agglomeration of carbon nanotubes (CNTs) on the tensile mechanical properties of CNT-reinforced epoxy resin (EP) nanocomposites through finite element simulation analysis. The results demonstrated that, for composites with low CNT mass fractions (0.5 wt%), simulations assuming uniform filler distribution accurately predicted tensile strength and elastic modulus. Conversely, for composites with higher CNT mass fractions (1.5 wt%), the modified analysis method incorporating CNT agglomeration effects achieved superior precision in mechanical property predictions, exhibiting enhanced accuracy and reliability.

F. Jeyranpour et al. [129] employed molecular dynamics (MD) simulations to investigate the thermodynamic and mechanical properties of epoxy networks derived from the reaction of DGEBA with two curing agents, TETA and DETDA, at varying crosslinking densities. The study revealed that, for the DGEBA/TETA system, a crosslinking density of 62.5% yielded the closest agreement between the glass transition temperature (Tg) calculated from density–temperature curves and experimental values, with Tg ranging from 385–395 °C. Furthermore, systematic analyses were conducted on the temperature-dependent variations in the coefficient of thermal expansion (CTE), elastic modulus, Poisson’s ratio, and density across different crosslinking levels. Comparative evaluation of the DGEBA/TETA and DGEBA/DETDA systems demonstrated that increasing crosslinking density enhanced Tg and mechanical performance, though these improvements plateaued at higher crosslinking densities. Overall, the DGEBA/TETA system exhibited superior mechanical properties, while the DGEBA/DETDA system consistently achieved higher Tg values under all tested conditions.

### 7.2. Machine Learning

With the rapid advancement of data science, machine learning (ML) techniques [130,131] have emerged as powerful tools for predicting the thermomechanical properties and fracture characteristics of epoxy composites. Machine learning models trained on experimental or simulated datasets can be utilized for virtual screening of epoxy formulations, optimization of composite designs, and accelerated prediction of key performance metrics including fracture toughness, modulus, and glass transition temperature [132,133,134].

**Figure 9:**
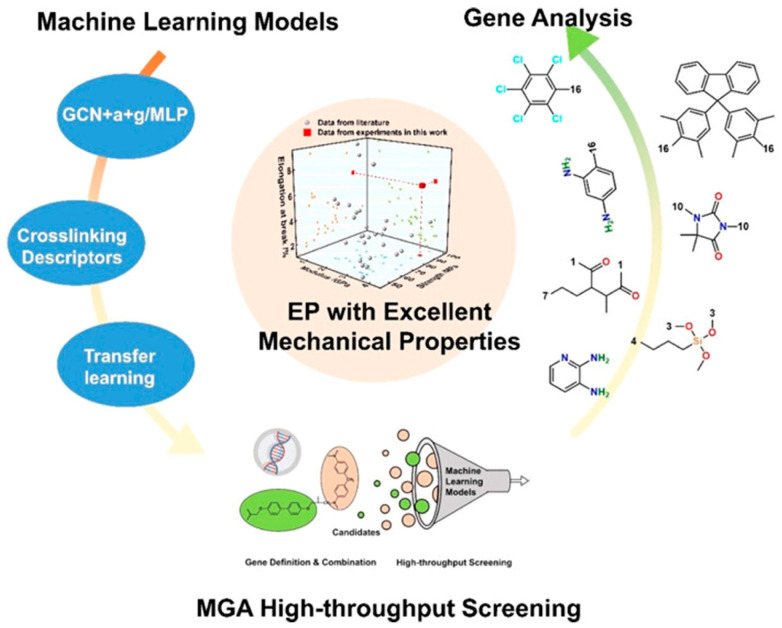
MGA High-Throughput Screening (MGA-HTS) [135].

Hu et al. [135] proposed a machine-learning-enabled materials genome approach (MGA) for rapid design of epoxy resin (EP) polymers with high modulus, high strength, and high toughness. By integrating attention-enhanced and gating-enhanced graph convolutional networks (GCN+a+g), multi-layer perceptrons (MLPs), classical gel theory, and transfer learning from small molecules to polymers, they developed predictive models to accurately forecast the mechanical properties of EPs. As illustrated in Figure 9, high-throughput screening across extensive chemical spaces successfully identified a series of EP candidates with outstanding comprehensive mechanical properties, whose performance was experimentally validated. Furthermore, critical genetic substructures influencing polymer mechanical behaviors were systematically analyzed, unveiling the microscopic mechanisms underlying high-performance EP design.

Jang et al. [136] established a predictive framework integrating molecular dynamics (MD) simulations and machine learning methodologies to accurately determine key physicochemical properties of epoxy resins, including density, coefficient of thermal expansion (CTE), glass transition temperature (Tg), and Young’s modulus. This methodology commenced with the generation of 789 distinct epoxy resin configurations via MD simulations, followed by the extraction of molecular descriptors as input features for training machine learning models. The results demonstrated strong predictive accuracy across multiple material properties, highlighting the framework’s potential for accelerating rational material design and high-throughput screening of advanced epoxy systems.

Jin et al. [137] proposed a hybrid methodology integrating molecular dynamics (MD) simulations and machine learning (ML) for the rational design of high-performance epoxy resin systems. By generating training datasets through MD simulations and subsequently employing ML to predict the effects of compositional variations on material performance, this approach enabled systematic optimization of epoxy formulations. The study identified critical roles of key components: 4,4′-diaminodiphenyl sulfone (DDS) as a curing agent significantly enhanced glass transition temperature (Tg), Young’s modulus (E), and elongation at break (δ), while tetraglycidyl diamino diphenylmethane (TGDDM) ensured high Tg, E, and ultimate tensile strength (UTS). To further improve toughness, controlled incorporation of diglycidyl ether of bisphenol A (DGEBA) proved essential. Additionally, polyether sulfone (PES) demonstrated effectiveness as a toughening agent, enhancing fracture resistance without compromising modulus. The optimized epoxy system designed via this methodology achieved simultaneous performance enhancements: 17.3% increase in Tg, 15% improvement in E, 32.7% elevation in UTS, and 85.5% gain in δ, while maintaining balanced thermomechanical properties.

### 7.3. Summary

Computational modeling and machine learning (ML) demonstrate complementary advantages in epoxy resin research. Computational modeling techniques, such as molecular dynamics (MD) and finite element analysis (FEA), elucidate material micromechanisms rooted in physical principles, including crosslinked network evolution. In contrast, ML enables rapid performance prediction (e.g., fracture toughness) through data-driven pattern recognition, bypassing explicit mechanistic modeling.

## 8. Conclusions and Perspective

The rubber toughening method, thermoplastic resin toughening method, hyperbranched polymer toughening method, and flexible chain segment toughening method are all effective strategies for enhancing the toughness of epoxy resins. Key factors for evaluating the effectiveness of these toughening methods include the degree of toughness improvement, processability, and the impact on other material properties. Rubber toughening significantly improves the impact resistance and ductility of epoxy resins at a low cost. However, this approach has its limitations, particularly the tendency of rubber to reduce the thermal resistance, aging resistance, and thermal stability of the epoxy resin, which may be limiting factors in practical applications.

In contrast, the use of thermoplastic resins to toughen epoxy resins can effectively address these issues. Thermoplastic resins not only enhance the toughness of epoxy resins but can also improve their modulus and thermal resistance to some extent. The main drawback is the poor solubility and flowability of thermoplastic resins, which can increase the viscosity of the cured system and adversely affect processability. To achieve epoxy resins with both excellent toughness and good processability, it is important to carefully consider the type and amount of thermoplastic resin used, as well as to optimize its distribution and interactions within the matrix.

Additionally, hyperbranched polymers, as a novel toughening agent, can improve toughness with minimal impact on other properties of the epoxy resin. Although their synthesis is complex and costly, hyperbranched polymers remain a viable option when performance and cost considerations are both critical. The flexible chain segment toughening method, which involves modifying epoxy resins with flexible curing agents, offers a straightforward and practical toughening solution. While this method may affect the thermal resistance and tensile strength of the epoxy resin, it is generally easy to implement and has good practical applicability.

Furthermore, nanoparticle toughening has emerged as a promising approach, significantly enhancing both the toughness and strength of epoxy resins, thereby broadening their application potential. The development of hybrid epoxy nanocomposites further enables the integration of high performance and multi-functionality in epoxy-based materials. Nevertheless, challenges such as nanoparticle aggregation—attributed to their high surface activity and large specific surface area—result in uneven dispersion within the resin matrix, ultimately diminishing toughening efficiency.

However, despite the development of various epoxy resins based on different toughening mechanisms, and the significant progress made in enhancing their toughness, several challenges remain unresolved. The preparation processes for most toughened epoxy resins are complex, leading to high production costs, which in turn limit their widespread adoption in large-scale industrial applications.

Notably, computational modeling and machine learning (ML) technologies have emerged as transformative paradigms for the performance optimization and prediction of epoxy resins. Computational modeling techniques, such as molecular dynamics (MD) and finite element analysis (FEA), elucidate material micromechanisms through fundamental physical principles, including crosslinking dynamics and stress distribution at molecular/interfacial scales. Concurrently, ML enables rapid property prediction (e.g., fracture toughness, thermal stability) via data-driven correlations, circumventing explicit physical modeling. Collectively, these approaches significantly reduce reliance on trial-and-error experimentation while enhancing the efficiency of toughened epoxy resin development.

Looking forward, the development of more efficient and effective toughening strategies for epoxy resins remains a critical area of research. Future work should focus on the following aspects:

1. Advanced Material Design: Exploring new molecular designs and hybrid toughening systems that combine the strengths of multiple toughening agents while minimizing their weaknesses will be crucial. This could involve the development of novel core–shell structures, multi-functional nanoparticles, or bio-inspired materials that offer enhanced toughness without compromising other critical properties. Furthermore, existing research has primarily focused on evaluating the static mechanical properties of toughened epoxy resins, often overlooking their performance under dynamic loading conditions. However, in real-world applications, epoxy resins frequently encounter complex stress states, necessitating a deeper investigation into their dynamic mechanical behavior. Additionally, only a limited number of studies have examined the changes in the mechanical properties of epoxy resins under extreme environments, such as high temperatures and high humidity. Understanding these changes is crucial for the practical application of these materials.
2. Optimization of Processing Techniques: The synthesis and processing conditions play a significant role in determining the final properties of toughened epoxy resins. Advances in processing technologies, such as controlled phase separation techniques and in situ polymerization methods, could lead to more uniform dispersion of toughening agents and better integration within the resin matrix.
3. Environmental and Economic Considerations: With the increasing focus on sustainability, research should also address the environmental impact and cost-effectiveness of toughening agents. The development of eco-friendly toughening agents derived from renewable sources, as well as the optimization of processes to reduce energy consumption and waste, will be essential for the broader adoption of these technologies.
4. Application-Specific Optimization: Tailoring toughening strategies to meet the specific requirements of various industrial applications, such as aerospace, automotive, and electronic, will be important. This could involve the customization of resin formulations to achieve the desired balance of properties, including toughness, thermal stability, electrical conductivity, and chemical resistance.
5. Computer modeling and machine learning exhibit distinct advantages in epoxy resin research. Through synergistic integration of these approaches—where simulation-generated data augment ML training while ML-driven acceleration optimizes modeling parameters—the streamlined development of toughened epoxy resins can be effectively achieved.

By addressing these challenges and opportunities, future research can further enhance the performance of epoxy resins, expanding their use in high-performance applications and contributing to the development of more durable, reliable, and sustainable materials.

## Figures and Tables

**Table 1:** Comparison of the advantages and disadvantages of various toughening methods.

Method	Advantages	Disadvantages
Rubber Toughening [4,13]	Modest increase in toughness; low cost	Poor compatibility with epoxy resin; significant reduction in thermal and mechanical properties of epoxy resin
Thermoplastic Resin Toughening [14,15]	Significant increase in toughness; does not affect modulus or heat resistance; low cost	Increased viscosity of the cured system, leading to reduced solubility; slight reduction in thermal and mechanical properties
Nanoparticle Toughening [16]	Modest increase in toughness; improves fracture toughness and tensile strength; slight improvement in thermal and mechanical properties	Poor dispersion of nanoparticles; high cost
Hyperbranched Polymer Toughening [17,18]	Significant increase in toughness; slight improvement in mechanical properties	Complex synthesis and expensive
Flexible Chain Segment Toughening [19,20]	Significant increase in toughness; optimized performance	Reduction in heat resistance and tensile strength
